# Therapeutic Potential of *Lactiplantibacillus plantarum* FB091 in Alleviating Alcohol-Induced Liver Disease through Gut-Liver Axis

**DOI:** 10.4014/jmb.2407.07051

**Published:** 2024-08-09

**Authors:** Soo-Jeong Lee, Jihye Yang, Gi Beom Keum, Jinok Kwak, Hyunok Doo, Sungwoo Choi, Dong-Geun Park, Chul-Hong Kim, Hyeun Bum Kim, Ju-Hoon Lee

**Affiliations:** 1 Department of Food and Animal Biotechnology, Department of Agricultural Biotechnology, Research Institute of Agriculture and Life Sciences, Center for Food and Bioconvergence, Seoul National University, Seoul 08826, Republic of Korea; 2 Department of Animal Biotechnology, Dankook University, Cheonan 31116, Republic of Korea; 3 Binggrae Company, Namyangju 12253, Republic of Korea

**Keywords:** Alcohol-induced liver disease, probiotics, gut-liver axis, gut microbiota, colon inflammation

## Abstract

Alcoholic liver disease (ALD) poses a significant global health burden, often requiring liver transplantation and resulting in fatalities. Current treatments, like corticosteroids, effectively reduce inflammation but carry significant immunosuppressive risks. This study evaluates *Lactiplantibacillus plantarum* FB091, a newly isolated probiotic strain, as a safer alternative for ALD treatment. Using an in vivo mouse model, we assessed the effects of *L. plantarum* FB091 on alcohol-induced liver damage and gut microbiota composition. Alcohol and probiotics administration did not significantly impact water/feed intake or body weight. Histopathological analysis showed that *L. plantarum* FB091 reduced hepatocellular ballooning and inflammatory cell infiltration in liver tissues and mitigated structural damage in colon tissues, demonstrating protective effects against alcohol-induced damage. Biomarker analysis indicated that *L. plantarum* FB091 decreased aspartate aminotransferase levels, suggesting reduced liver damage, and increased alcohol dehydrogenase activity, indicating enhanced alcohol metabolism. Additionally, cytokine assays revealed a reduction in pro-inflammatory TNF-α and an increase in anti-inflammatory IL-10 levels in colon tissues of the *L. plantarum* FB091 group, suggesting an anti-inflammatory effect. Gut microbiota analysis showed changes in the *L. plantarum* FB091 group, including a reduction in Cyanobacteria and an increase in beneficial bacteria such as *Akkermansia* and *Lactobacillus*. These changes correlated with the recovery and protection of liver and colon health. Overall, *L. plantarum* FB091 shows potential as a therapeutic probiotic for managing ALD through its protective effects on liver and colon tissues, enhancement of alcohol metabolism, and beneficial modulation of gut microbiota. Further clinical studies are warranted to confirm these findings in humans.

## Introduction

Alcohol consumption has been identified as a causal risk factor for over 200 diseases, injuries, and other health conditions [1- 3]. In particular, noncommunicable diseases such as liver cirrhosis, cardiovascular disease, and mental and behavioral disorders, including alcohol dependence, are strongly associated with alcohol consumption [4]. Liver is particularly vulnerable to alcohol-related disease as it is the primary organ for alcohol metabolism [5]. Therefore, chronic alcohol intake can lead to alcoholic liver disease (ALD) including fatty liver disease, alcoholic hepatitis, and liver fibrosis or cirrhosis [6]. According to the rising incidence rates of ALD, it has become the leading indication for liver transplantation and is responsible for more than half of liver-related deaths [7, 8]. Corticosteroids, such as prednisolone or prednisone, are the most common treatments for ALD, due to their anti-inflammatory effects [9, 10]. These medications act similarly to cortisol, a hormone released by the body in response to injury or stress. They work by inhibiting transcription factors like nuclear factor kappa B (NF-κB), thereby reducing levels of proinflammatory cytokines such as interleukin-8 (IL-8) and tumor necrosis factor-alpha (TNF-α) in the bloodstream. However, the use of corticosteroids remains controversial, due to their immunosuppressive effects, increasing the patients' susceptibility to bacterial, viral, and fungal infections [11-14]. For instance, the use of 5 mg of prednisolone is associated with an 11%, 30%, or 55% increased risk of microbial infection compared to non-users if taken for 1, 3, or 12 months, respectively [15]. These significant risks highlight the need for developing new and safe treatment methods for ALD.

Probiotics are live microorganisms that, when administered in adequate amounts, confer health benefits to the host by promoting a healthy balance of gut microbiota [16]. As an example, these beneficial bacteria help in maintaining the integrity of the gut barrier, modulating the immune system, and outcompeting pathogenic bacteria [17, 18]. Probiotics have been widely studied for their role in gastrointestinal health, but recent research has also highlighted their potential benefits in hepatic and systemic conditions, particularly through the gut-liver axis, which represents a critical bidirectional relationship between the gut microbiota and liver function [19]. Liver is exposed to microbial products and metabolites from the gut microbiota through the portal vein, which can influence liver health and disease. The imbalance of gut microbiota could affect liver inflammation and fibrosis through the modulation of immune responses and the production of harmful metabolites [20, 21]. Furthermore, this imbalance in gut microbiota has been suggested to be associated with the progression of liver diseases, including ALD [22]. Therefore, re-balance of gut microbiota composition by supplementation of specific probiotics could be one of important treatment to alleviate ALD.

In previous preclinical studies, the administration of probiotics has demonstrated beneficial effects on liver diseases such as ALD via gut-liver axis [23-25]. For instance, ALD-induced rats administered *Lactiplantibacillus plantarum* B7 showed reduction of serum alanine aminotransferase (ALT; 0.61-fold reduction) and aspartate aminotransferase (AST; 0.52-fold reduction) levels compared to the ALD group. Because ALT and AST enzymes are related to liver damage or inflammation, their reductions in the blood suggest potential protective effect of the probiotics against liver damage, implying on the improvement in liver health. In addition, *L. plantarum* B7 treated group also exhibited reduced hepatocyte ballooning in liver histopathology, demonstrating attenuated alcohol-induced liver injury. Subsequent microbiome analysis in this B7-treated group showed increase of beneficial bacteria including *Bifidobacterium* and *Akkermansia* but their decrease in the gut microbiota of ALD group. This indicates that the intake of probiotics not only mitigated liver damage in ALD, but also positively altered the gut microbiota [26]. In other study, the beneficial effects of *Lactobacillus rhamnosus* GG (LGG) were investigated in ALD [27]. The supplementation of LGG decreased TNF-α, Toll-like receptors (TLRs), CYP2E1, and nuclear factor erythroid 2-related factor 2 (Nrf2) in mRNA levels, suggesting the reduction of hepatic inflammation and dysfunction [27]. In addition, gut microbiome analysis revealed that decrease of *Bacteroidetes* and *Firmicutes* but increase of *Proteobacteria* and *Actinobacteria*, highlighting the crucial role of microbiota in the gut-liver axis and the beneficial effects of LGG in ALD [28]. Therefore, through its effects on the gut-liver axis, probiotics could be a potential bioagent or alternative supplement for control and alleviation of ALD. However, the exact mechanisms for ALD alleviation via the gut-liver axis by probiotics supplementation remain to be elucidated.

In this study, a new probiotic strain, *L. plantarum* FB091, was isolated and its protection effect for alcohol-induced ALD was evaluated. To elucidate the therapeutic potential for ALD and gut microbiota modulation effect by FB091, in vivo feeding study and microbiome analysis were performed and analyzed. The findings in this study could offer new insights into managing and preventing alcoholic liver disease by supplementation of selected probiotics.

## Materials and Methods

### Bacterial Strain and Growth Condition

*L. plantarum* FB091 was previously isolated and stored in bacterial culture collection in Binggrae (Republic of Korea). *L. plantarum* ATCC 8014 was selected as a control strain. All *L. plantarum* strains were cultivated in de Man-Rogosa-Sharpe (MRS; Difco, USA) medium at 37°C for 24 h under anaerobic condition using BBL anaerobic GasPak jar system (BD, USA).

### In Vivo Mouse Feeding Study

**Fig. 1:**
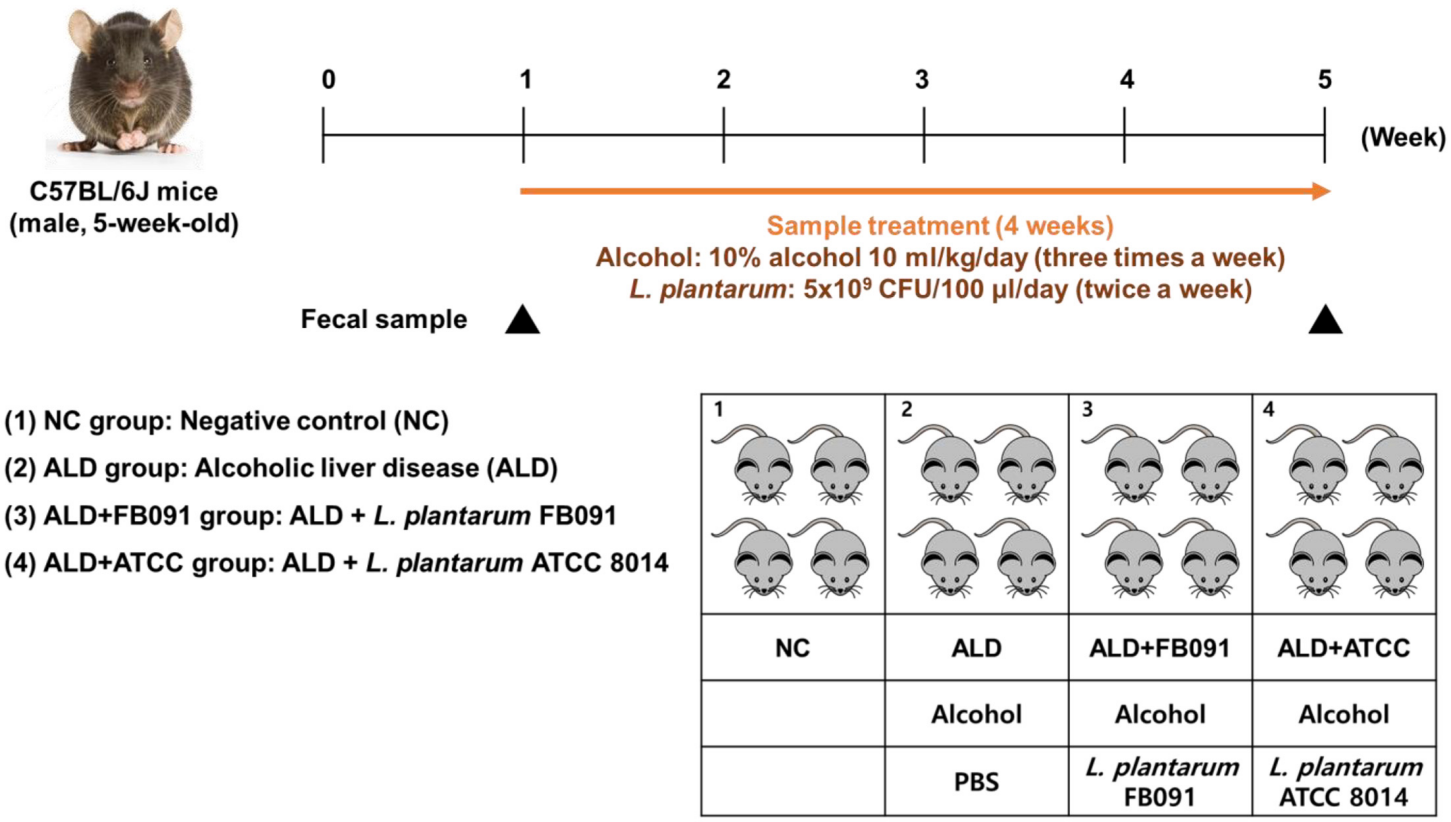
Experimental procedure of mouse feeding study with a one-week adaptation period and four-week feeding period. Water/feed intake and body weight were measured every week. Fecal samples were collected at week 1 and 5. NC: negative control, ALD: mouse feeding group with alcohol, ALD+FB091: mouse feeding group with alcohol and *L. plantarum* FB091, ALD+ATCC: mouse feeding group with alcohol and *L. plantarum* ATCC 8014.

Overall mouse feeding study was described in Fig. 1. Male C57BL/6J mice (5-week-old) were obtained from RaonBio (Republic of Korea) and acclimatized to laboratory conditions consisting of a 12:12-h light–dark cycle, 24°C, and 55% humidity for one week. Four mice were randomly picked and grouped into one of the following four groups: NC (negative control group), ALD (10% alcohol 10 ml/kg), ALD+FB091 (10% alcohol 10 ml/kg with *L. plantarum* FB091), and ALD+ATCC (10% alcohol 10 ml/kg with *L. plantarum* ATCC 8014). Alcohol and probiotics were administered via oral gavage using an oral zonde (3.8 cm/24G; RaonBio) attached to a 1 ml syringe. The two *L. plantarum* strains were administered twice a week (Tuesday and Thursday) at a concentration of 5 × 10^9^ CFU in 100 μl of 1X PBS buffer, while the ALD group received only 100 μl of 1X PBS. 10% alcohol was administered three times a week (Monday, Wednesday, and Friday) to all groups except NC group. After a one-week adaptation period, this in vivo mouse feeding study continued for four weeks. All experiments with the mice were approved by the Institutional Animal Care and Use Committee of Dankook University (Cheonan, Republic of Korea; approval no. DKU-22-072) and conducted in accordance with Care and Use of Laboratory Animals guidelines.

### Water and Feed Intake and Body Weight

Water and feed intake for each mouse were monitored every 3 or 4 days throughout the study. Water intake was measured using graduated water bottles, and feed intake was measured by weighing the feed provided and the leftover. Mice were weighed during the measurements of water and feed intake each week.

### Histopathology Analysis

Liver and colon tissues were collected and fixed in 10% neutral-buffered formalin. Subsequent sample preparation and staining were performed by KP&T Co. (Republic of Korea); The tissues were then embedded in paraffin, sectioned at 5 μm thickness, and stained with hematoxylin and eosin (H&E) for histological examination. The stained liver and colon sections were examined under a light microscope (Leica, Germany) for signs of steatosis, inflammation, and fibrosis.

### Hepatic Damage and Alcohol Metabolism-Related Enzyme Activity Assay

To investigate hepatic damage and alcohol metabolism, the activities of aspartate aminotransferase (AST), alcohol dehydrogenase (ADH), and aldehyde dehydrogenase (ALDH) were measured. Liver tissues were weighed and homogenized in EzRIPA lysis buffer (Atto, Japan) at a 1:10 ratio (w/v). The homogenates were then centrifuged at 14,000 ×*g* for 15 min at 4°C. The supernatant was collected, and protein concentrations were determined using the Pierce BCA Protein Assay Kit (Thermo Fisher Scientific, USA). Hepatic levels of AST, ADH, and ALDH were quantified using specific assay kits: the Nori Mouse Aspartate Transaminase ELISA Kit (Genorise Scientific, USA) for AST, the Alcohol Dehydrogenase Activity Assay Kit (Sigma-Aldrich, USA) for ADH, and the Aldehyde Dehydrogenase Activity Colorimetric Assay Kit (Sigma-Aldrich) for ALDH. All assays were performed according to the manufacturers’ protocols, and enzyme activities were determined by measuring the change in absorbance at 450 nm.

### Cytokine Assay

To evaluate the inflammation level in the gut, pro-inflammatory cytokine TNF-α and anti-inflammatory cytokine IL-10 in colon tissue samples were measured using EzWay Mouse ELISA Kits (Koma Biotech, Republic of Korea). Colon tissues were homogenized in EzRIPA lysis buffer (Atto) at a 1:10 ratio (w/v), followed by centrifugation at 14,000 ×*g* for 15 min at 4°C. The supernatant was collected, and protein concentrations were determined using the Pierce BCA Protein Assay Kit (Thermo Fisher Scientific). ELISA assays were conducted according to the manufacturer's protocols, and cytokine levels were determined by measuring absorbance at 450 nm.

### Microbiome Analysis

Stool samples from each mouse were collected on the first day of Week 1 and the last day of Week 5, then stored at -80°C until use. DNA was extracted from the stool samples using the QIAamp DNA Stool Mini Kit (Qiagen, USA) following the manufacturer's protocol. The quality of the extracted DNA was assessed with a NanoDrop 2000 spectrophotometer (Thermo Fisher Scientific), and the concentration was determined using Qubit dsDNA HS Assay Kits on a Qubit 4.0 Fluorometer (Thermo Fisher Scientific). The 16S rRNA gene was amplified and barcoded using the SQK-16S024 kit (Oxford Nanopore Technologies, UK), with the universal primers of 27F (5'-AGAGTTTGATCCTGGCTCAG-3') and 1492R (5'-GGTTACCTTGTTACGACTT-3') containing unique barcode sequences. Amplification was performed using LongAmp Hot Start PCR Master Mix (New England Biolabs, USA), and the PCR products were purified using AMPure XP beads (Beckman Coulter, USA). Barcoded libraries were pooled to a total of 50-100 fmoles in 10 μl of 10 mM Tris-HCl (pH 8.0) and sequenced on a Nanopore MinION Flow cell R9.4.1 (Oxford Nanopore Technologies), according to the manufacturer's instructions. Base-calling of raw reads was performed using the Guppy basecaller, and reads were demultiplexed and adapters trimmed using qcat [29, 30]. For each sample, sequence reads were normalized based on the copy number of the 16S rRNA gene in the respective bacterial genome and the total number of sequenced reads. Sequence reads were aligned and classified using the EMU software against a custom database that included sequences from the rrnDB v5.648 and NCBI 16S RefSeq databases [31-33]. Minimap2 was employed in EMU for read alignment, and an expectation-maximization approach, used as EMU's clustering method, was applied to estimate taxonomic abundance [34]. Data were visualized using RStudio with ggplot2 and the related packages [35].

### Statistical Analysis

All data are expressed as the mean ± SD. Statistical significance was assessed by Student’s *t*-test and one-way ANOVA followed by post-hoc Tukey’s test for multiple comparisons. IBM SPSS software version 26 (USA) was used to perform all statistical tests. A value of *p* < 0.05 was considered as statistically significant.

## Results

### Pathological Influence on Water/Feed Intake and Body Weight by ALD

**Fig. 2:**
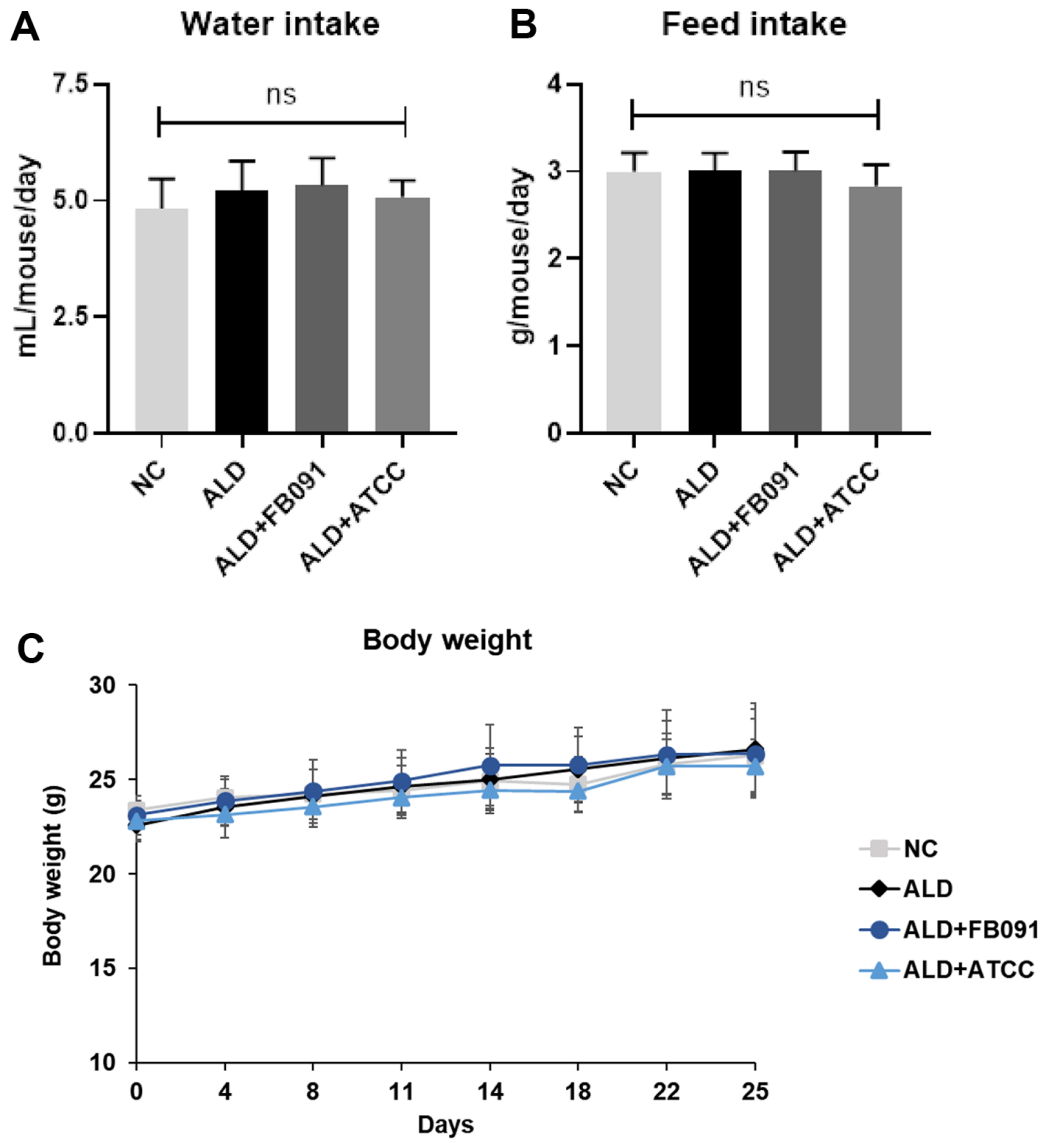
Average intake of water and feed, and changes in body weight. (**A**) Average of water intake every day in each group. (**B**) Average of feed consumption amount every day in each group. (**C**) Body weight changes for weeks 0-5 in each group. Error bars present the standard deviations of four replicated (*n* = 4 male mice) in each group. One-way ANOVA followed by Tukey’s post-hoc analysis (*p* < 0.05; ns, not significant).

Intakes of water and feed were measured and showed no significant difference among the four groups (Fig. 2A and 2B). The average water intake (ml/mouse/day) was 4.84 for NC, 5.21 for ALD, 5.33 for ALD+FB091, and 5.07 for ALD+ATCC. The average feed intake (g/mouse/day) was 3.00 for NC, 3.00 for ALD, 3.01 for ALD+FB091, and 2.84 for ALD+ATCC. Based on this, it is suggested that alcohol and probiotics administration do not influence on the intake levels of water and feed.

Body weight was measured once a week throughout the study (Fig. 2C). The initial average body weights before feeding were 23.39 g for NC, 22.59 g for ALD, 23.11 g for ALD+FB091, and 22.80 g for ALD+ATCC. By the end of the study, the final average body weights had increased to 26.28 g for NC, 26.595 g for ALD, 26.375 g for ALD+FB091, and 25.69 g for ALD+ATCC. Similar to the water and feed intake, this result suggests that alcohol and probiotics administration do not influence on the weight gain of test mice.

### Histopathological Analysis for Observation of Liver/Colon Damage and Inflammation

**Fig. 3:**
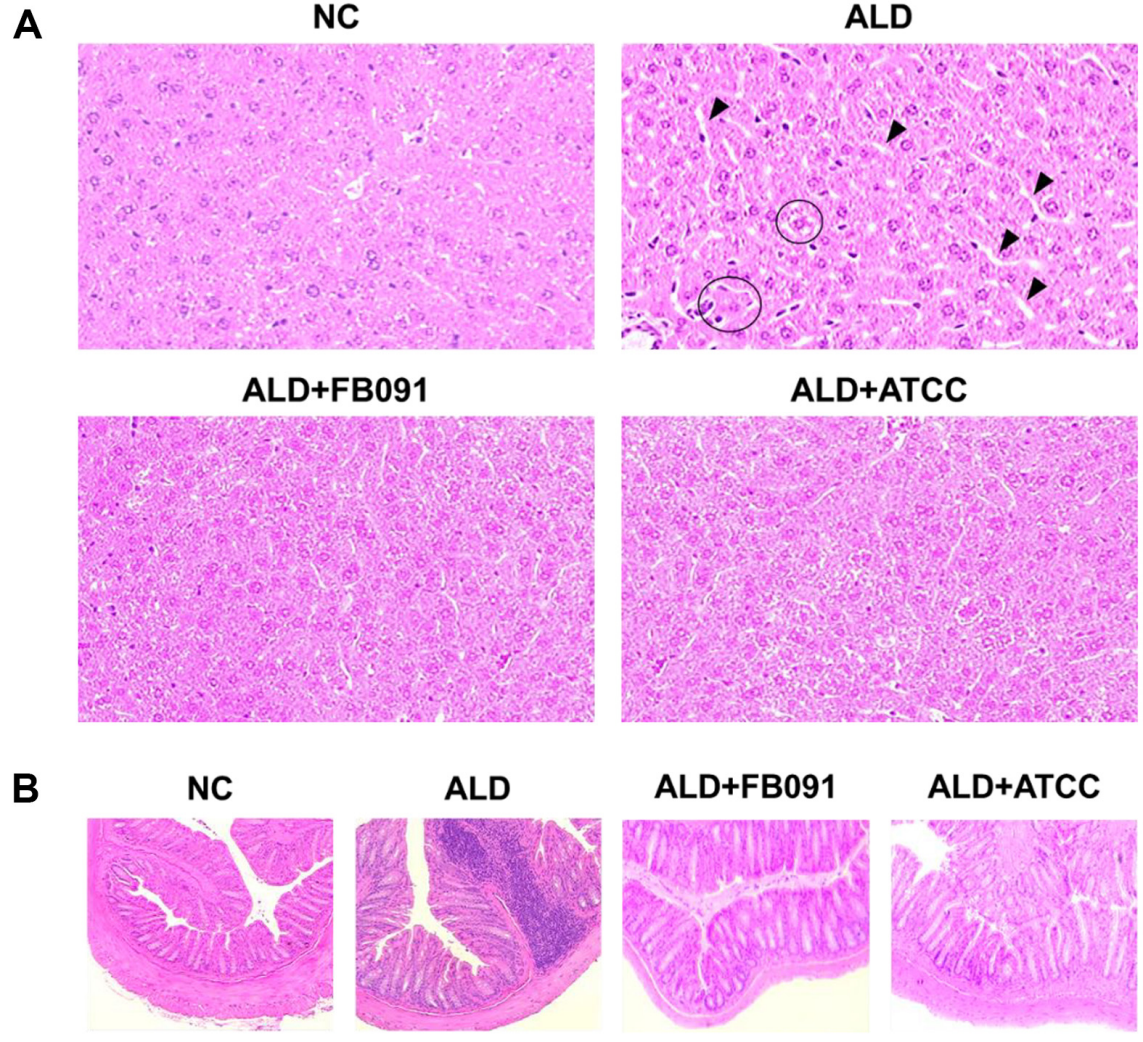
Histopathological analysis of the liver and colon. (**A**) Representative H&E staining of liver tissues. Circles indicate the hepatocellular ballooning and arrows indicate the hepatic cell infiltration. (**B**) Representative H&E staining of colon tissues. Magnification is 200×.

Histopathological analysis was performed with liver and colon tissues to confirm damage and inflammation of liver and colon structures, indicating no damage or inflammation in NC group (Fig. 3). In contrast, ALD group exhibited hepatocellular ballooning (marked by circles) and inflammatory hepatic cell infiltration (marked by arrows). Furthermore, distortion of the colon crypt structure was observed in ALD group, suggesting both liver/colon damage and inflammation (Fig. 3B). However, ALD+FB091 group showed a reduction in hepatocellular ballooning and inflammatory hepatic cell infiltration, with no structural damage of colon crypts. In addition, ALD+ATCC group displayed a similar pattern in the liver to ALD+FB091 group, but colon structure damage was observed. Therefore, while both FB091 and ATCC 8014 strains have damage protection effects in liver tissues, only FB091 has damage protection effects in colon tissues.

### Levels of Hepatic Damage Biomarker and Alcohol Metabolism-Related Enzymes

**Fig. 4:**
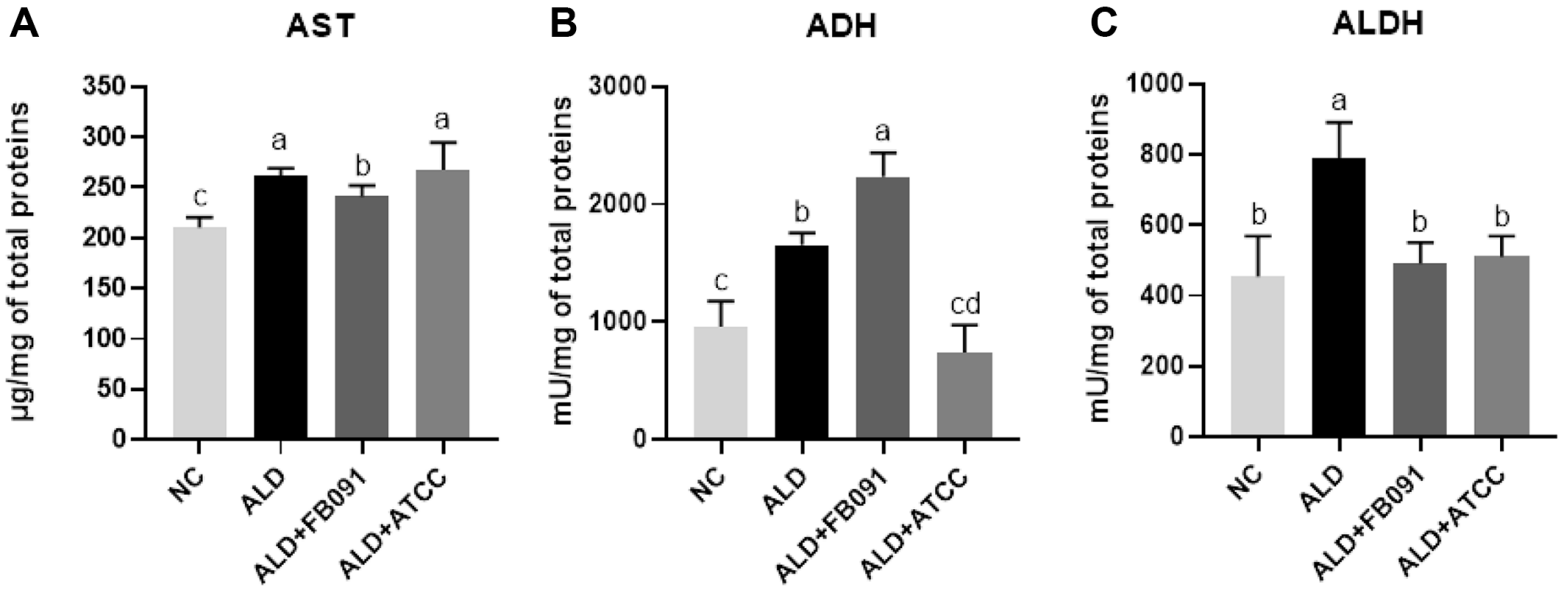
Hepatic damage biomarker levels and alcohol metabolism-related enzyme activities in all mouse groups. (**A**) Aspartate aminotransferase (**AST**) levels of liver tissues. (**B**) Alcohol dehydrogenase (**ADH**) activities of liver tissues. (**C**) Aldehyde dehydrogenase (**ALDH**) activities of liver tissues. Error bars present the standard deviations of four replicated (*n* = 4 male mice) in each group. One-way ANOVA followed by Tukey’s *post-hoc* analysis, and bars with different letters indicate significant differences at *p* < 0.05.

Hepatic damage and alcohol metabolism were assessed by measuring three key biomarkers: AST, ADH, and ALDH. AST is an indicator for alcohol-induced liver damage, while ADH and ALDH metabolize alcohol in the liver. ADH converts ethanol to acetaldehyde, and ALDH further converts acetaldehyde to the less toxic acetic acid (Fig. 4).

In ALD group, the AST level (261.03 ± 8.07 μg/mg total proteins) was 1.24-fold higher than that of NC group (210.00 ± 10.29 μg/mg total proteins), indicating increase of liver damage. However, ALD+FB091 group (240.67 ± 11.21 μg/mg total proteins) exhibited a 0.92-fold reduction in AST levels compared to ALD group, while ALD+ATCC group (267.40 ± 27.12 μg/mg) showed no change. Therefore, lowering of AST levels in the ALD+FB091 group suggests reduction of liver stress or damage, compared to the ALD group.

In addition to AST, ADH levels were determined in all four test groups. The ADH level of ALD group (1654.20± 101.35 mU/mg total proteins) was 1.73-fold higher than that of NC group (958.18 ± 218.06 mU/mg total proteins). Interestingly, ALD+FB091 group (2233.41 ± 205.78 mU/mg total proteins) showed the highest ADH activity with 2.33-fold and 1.35-fold increase, compared to NC group and ALD group, respectively, suggesting that ALD+FB091 group has the highest bioconversion activity of alcohol to acetaldehyde. However, ALD+ATCC group (738.40 ± 234.69 mU/mg total proteins) exhibited the lowest activity. Based on these results, FB091administration could be helpful for alcohol degradation while ATCC 8014 administration might be not helpful.

For ALDH activity, ALD group (788.51 ± 102.87 mU/mg total proteins) showed the highest activity. However, other three test groups revealed similar ALDH activities without significant difference: NC (454.85 ± 114.30 mU/mg total proteins), ALD+FB091 (491.17 ± 59.10 mU/mg total proteins), and ALD+ATCC (519.83 ± 58.16 mU/mg total proteins). These results suggest that FB091 or ATCC 8014 administration do not affect bioconversion activity of acetaldehyde to acetic acid like NC group.

Based on these results, FB091 administration is effective for alcohol degradation and bioconversion to non-toxic compound by control or regulation of alcohol metabolism enzymes: lowering of AST activity, induction of ADH activity, and maintaining of ALDH activity. This effective enzyme regulation by FB091 administration may be useful for protection and recovery of damage in liver and colon tissues from alcohol intake.

### Immune Regulation of *L. plantarum* FB091 in the Mouse Colon

**Fig. 5:**
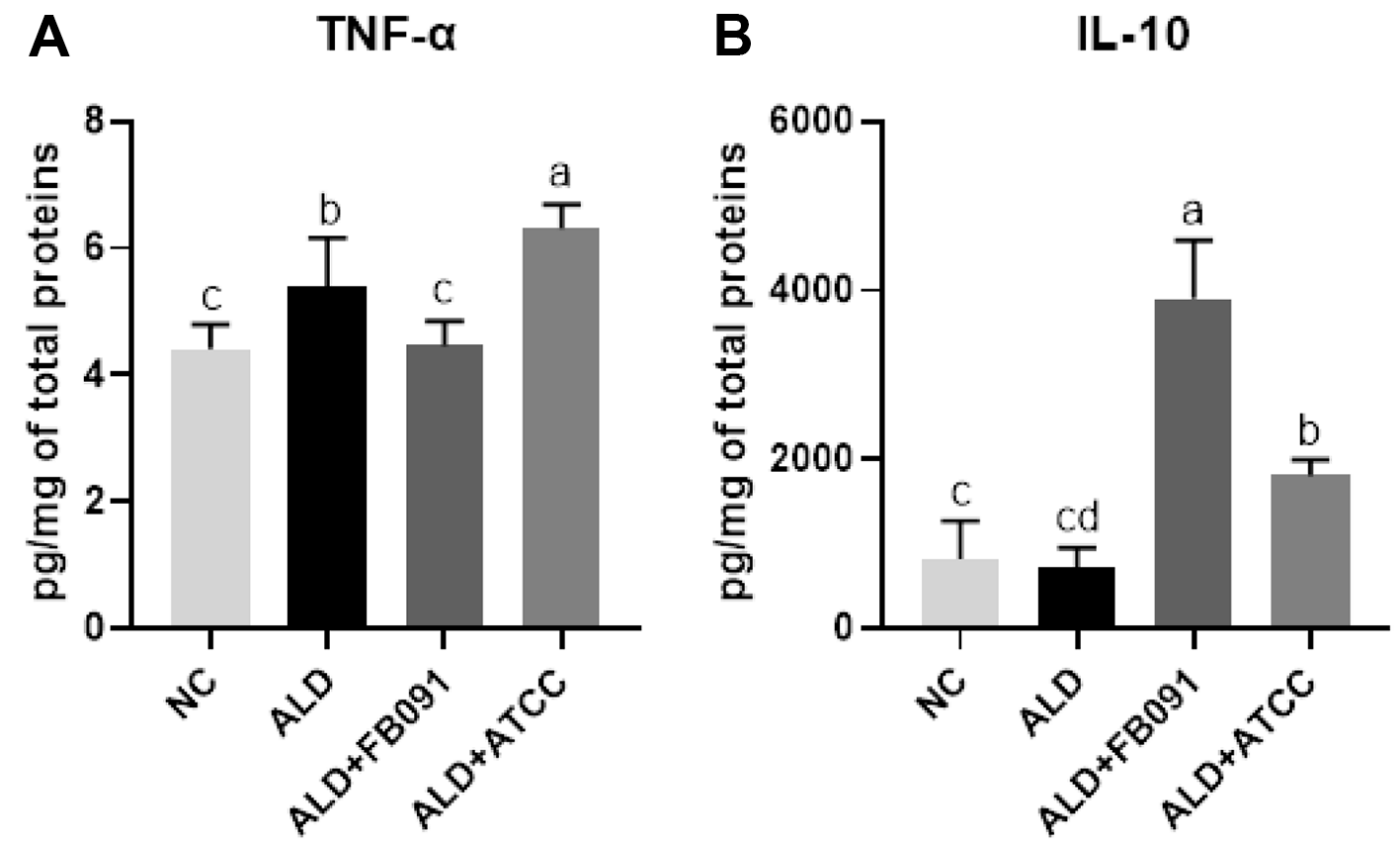
Inflammatory cytokine levels of colon in all mouse groups. (**A**) Tumor necrosis factor (**TNF**)-α levels, (**B**) Interleukin (**IL**)-10 levels. Error bars present the standard deviations of four replicated (*n* = 4 male mice) in each group. Oneway ANOVA followed by Tukey’s *post-hoc* analysis, and bars with different letters indicate significant differences at *p* < 0.05.

Cytokine assay with colon tissues was performed to determine the colon inflammation by measuring the pro-inflammatory cytokine TNF-α and the anti-inflammatory cytokine IL-10 (Fig. 5). In ALD group, the TNF-α level (5.38 ± 0.79 pg/mg total proteins) was 1.22-fold higher than that of NC group (4.41 ± 0.39 pg/mg total proteins), indicating increase of colon inflammation. However, ALD+FB091 group (4.46 ± 0.38 pg/mg total proteins) exhibited a 0.83-fold reduction in TNF-α level, compared to ALD group, while ALD+ATCC group (6.32 ± 0.37 pg/mg) showed 1.17-fold increase. Therefore, the reduction of TNF-α levels in ALD+FB091 group suggests decrease in colon inflammation compared to the ALD group, while ALD+ATCC group showed no effect on reduction of colon inflammation.

In addition to TNF-α, IL-10 levels were determined in all four test groups. The IL-10 level in ALD group (722.94± 231.46 pg/mg total proteins) was 0.89-fold lower than that in the NC group (815.14 ± 456.10 pg/mg total proteins). Interestingly, ALD+FB091 group (3904.36 ± 690.50 pg/mg total proteins) showed the highest IL-10 level, with 4.79-fold and 5.40-fold increase, compared to the NC group and the ALD group, respectively. In addition, ALD+ATCC group (1810.17 ± 189.54 pg/mg total proteins) exhibited a 2.22-fold increase over the NC group and a 2.50-fold increase over the ALD group. Based on these results, both FB091 and ATCC 8014 administration could reduce the colon inflammation, but FB091 is probably more effective.

### Diversity and Compositional Changes of the Gut Microbiota

**Fig. 6:**
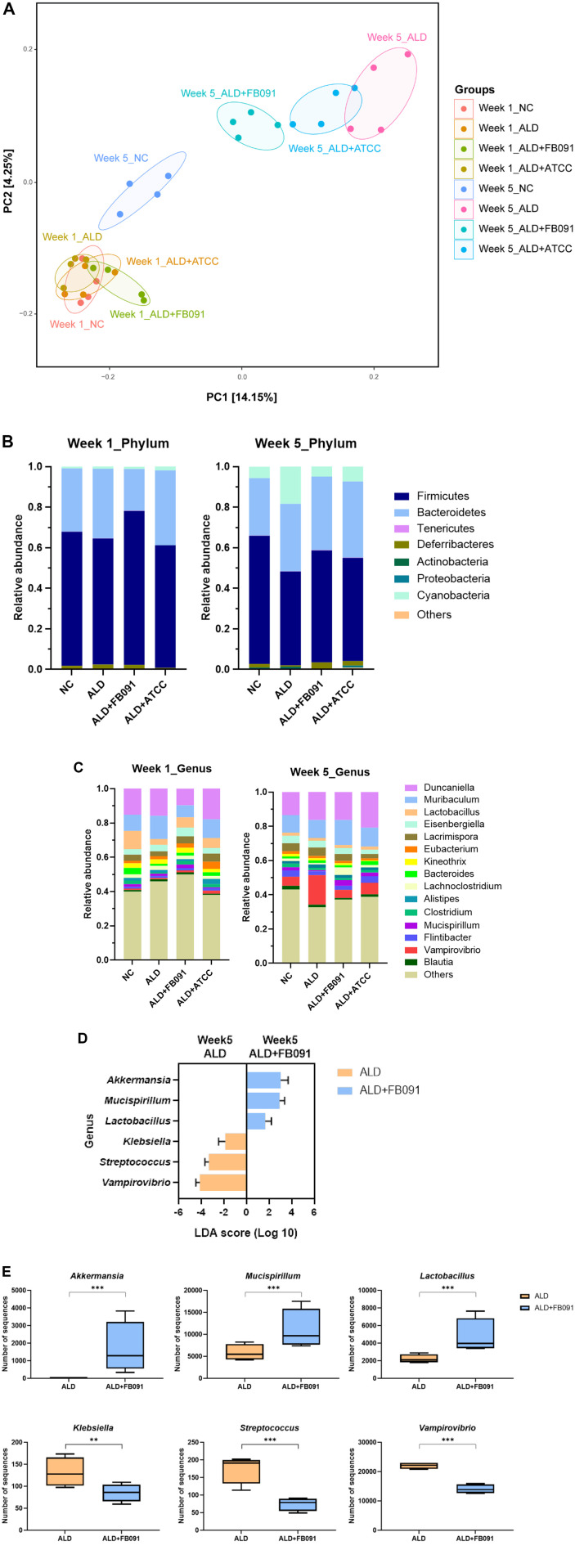
Microbiome analysis of fecal samples from the in vivo mouse model of all groups. (**A**) β-diversity plot of each group at weeks 1 and 5 using jaccard distance. (**B**) Gut microbiota composition at phylum level. (**C**) Gut microbiota composition at genus level. (**D**) Differential abundance analysis in genus level using LEfSe. (**E**) Boxplot of comparative composition analysis of *Akkermansia*, *Mucispirillum*, *Lactobacillus*, *Klebsiella*, *Streptococcus*, and *Vampirovibrio* at week 5 in the ALD and ALD+FB091 groups.

Fecal samples were collected from four groups (NC, ALD, ALD+FB091, and ALD+ATCC) at Weeks 1 and 5. The β-diversity analysis revealed no significant differences among these groups at Week 1. However, by Week 5, the gut microbiota composition in all groups had altered and diverged from their Week 1 counterparts (Fig. 6A). The NC group at Week 5 exhibited minimal changes, whereas the ALD group (alcohol administration only) showed substantial divergence from Week 1. Notably, the ALD+FB091 and ALD+ATCC groups (alcohol and probiotics administration) converged towards the NC group at Week 5, with ALD+FB091 displaying greater similarity to the NC group. This suggests that probiotic administration may effectively aid in the recovery of gut microbiota composition disrupted by alcohol administration, with FB091 potentially offering better recovery (Fig. 6A).

To further investigate the liver protective effects of probiotics, the compositional changes in gut microbiota due to alcohol administration were monitored. Comparative analysis at the phylum level between Week 1 and Week 5 revealed a similar ratio of *Bacteroidetes* and *Firmicutes* (Fig. 6B). Interestingly, Cyanobacteria were detected at Week 5 and significantly increased in the ALD group, suggesting a potential association between alcohol administration and the rise in Cyanobacteria. However, the proportions of Cyanobacteria in the ALD+FB091 and ALD+ATCC groups were reduced, similar to the levels observed in the NC group. This finding suggests a possible recovery of gut microbiota composition with probiotic administration. Notably, previous studies have linked a higher abundance of Cyanobacteria in gut microbiota to an increased risk of liver diseases, such as hepatocellular carcinoma [36]. Additionally, some Cyanobacteria produce microcystins, hepatotoxins that are potent promoters of liver tumors [37]. Therefore, the increase of Cyanobacteria in gut microbiota may be correlated with adverse effects on liver health.

The compositional changes at the genus level between Week 1 and Week 5 mirrored those observed at the phylum level. Notably, *Vampirovibrio* significantly increased in the ALD group at Week 5 (Fig. 6C). This increase is particularly interesting as *Vampirovibrio* belongs to the phylum Cyanobacteria, suggesting it may be responsible for the observed rise in Cyanobacteria at the phylum level. Similar to the phylum-level analysis, the proportion of *Vampirovibrio* was reduced in the ALD+FB091 and ALD+ATCC groups, aligning with the levels seen in the NC group, indicating the possible recovery effects by probiotics administration (Fig. 6C). It has been reported that *Vampirovibrio* is a predatory bacterium that preys on other bacteria, thereby influencing the composition and diversity of the microbial community [36]. Recent research has elucidated the pathogenic mechanism of *Vampirovibrio*, which involves using chemotaxis and a type IV secretion system to locate and attach to its prey, transferring plasmid DNA and hydrolytic enzymes to facilitate replication [38]. Since its first detection in human microbiota in 2017, *Vampirovibrio* has been frequently found in high abundance in cystic fibrosis patients [39]. However, its functional role and significance in the gut microbiota remain poorly understood, and its correlation with alcohol administration is still unknown.

The Linear Discriminant Analysis Effect Size (LEfSe) between the ALD and ALD+FB091 groups at Week 5 was conducted to elucidate the differential abundance of specific genera in each group. This analysis highlighted group-specific genera: the ALD group exhibited higher abundances of *Klebsiella*, *Streptococcus*, and *Vampirovibrio*, while the ALD+FB091 group was enriched with *Akkermansia*, *Muscipirillum*, and *Lactobacillus* (Fig. 6D). The dominant genera in each group were then quantified and compared, corroborating the LEfSe results (Fig. 6E). Therefore, this result confirmed again the reduction of *Vampirovibrio* by probiotics administration in the compositional analysis in genus level (Fig. 6C). The genera *Klebsiella* and *Streptococcus*, which were abundant in the ALD group, are associated with exacerbating liver disease and assessing its severity by increasing free radicals, leading to oxidative damage and necrosis of liver cells [40-42]. In contrast, the ALD+FB091 group showed high abundances of *Akkermansia* and *Lactobacillus*, which are known to be beneficial for gut health by enhancing intestinal barrier function and reducing inflammation [43, 44]. These findings suggest that FB091 administration beneficially modulates gut microbiota composition, increasing beneficial genera that support gut and liver health while reducing those associated with ALD pathology.

### Correlation Analysis of the Gut Microbiota and Biomarkers

To elucidate the correlation between gut microbiota at Week 5 and liver health, Spearman's correlation analysis was conducted. Two dominant bacterial sets from the gut microbiota were selected: *Akkermansia*, *Mucispirillum*, and *Lactobacillus* genera dominant in the ALD+FB091 group (Set I); *Klebsiella*, *Streptococcus*, and *Vampirovibrio* genera dominant in the ALD group (Set II) (Fig. 6D and 6E). Additionally, four biomarkers were selected: AST and ADH for liver health, and TNF-α and IL-10 for inflammation.

**Fig. 7:**
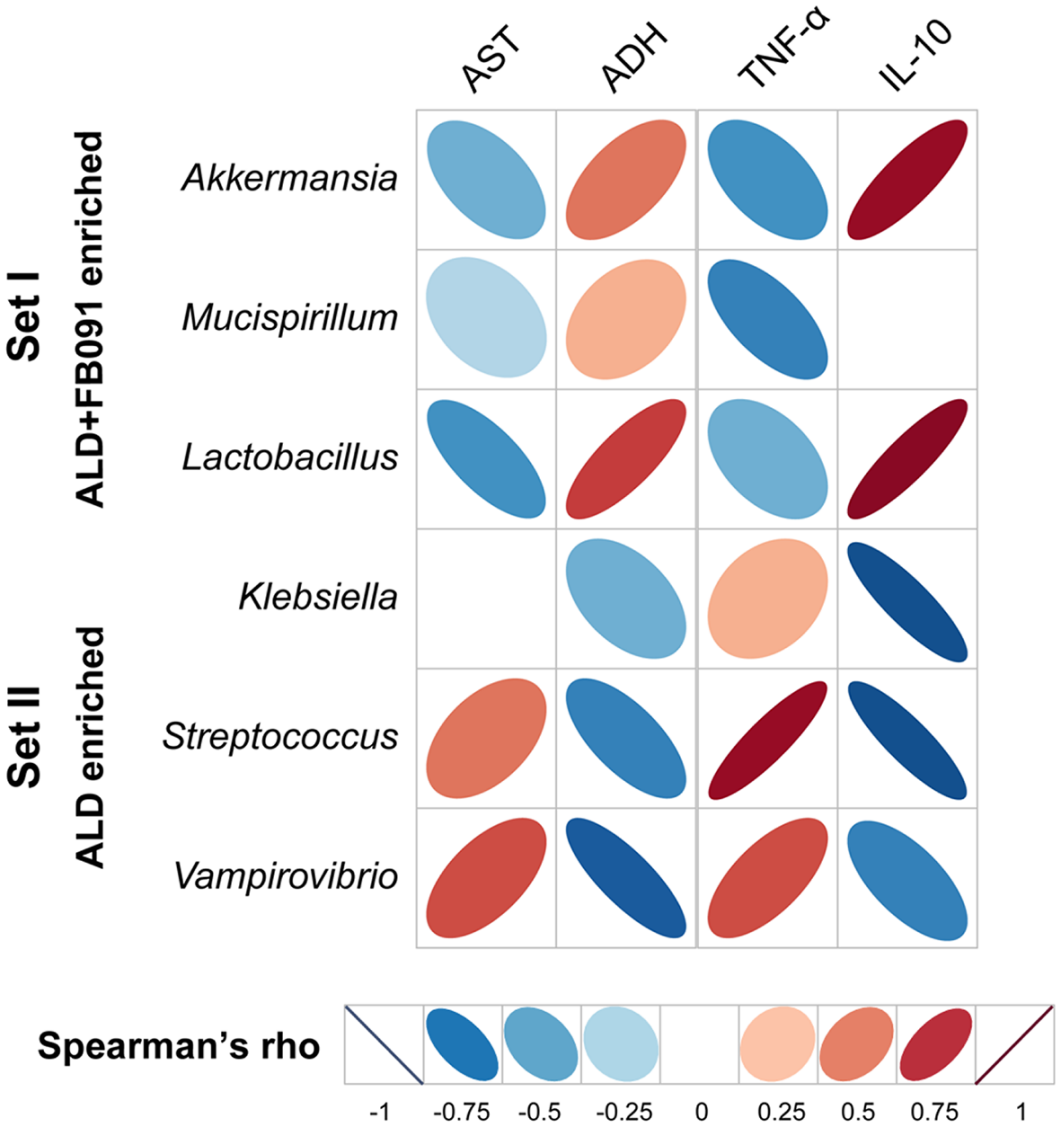
Spearman’s correlation analysis between gut microbiota and liver/colon damage or inflammation biomarkers. The biomarkers are correlated with specific genera.

For AST, a biomarker of liver damage, Set I bacteria showed a negative correlation, while Set II bacteria showed a positive correlation. This suggests that alcohol administration may be associated with liver damage, but FB091 administration may protect against or help recover from liver damage (Fig. 7). Moreover, ADH activity showed a positive correlation with Set I bacteria and a negative correlation with Set II bacteria, indicating that FB091 administration may facilitate the conversion of liver-toxic alcohol to aldehyde (Fig. 7). These results suggest that FB091 uptake may be effective for the recovery or protection of the liver from alcohol-induced damage.

Furthermore, the two inflammation-related biomarkers, pro-inflammatory cytokine TNF-α and anti-inflammatory cytokine IL-10, were monitored and compared between the ALD and ALD+FB091 groups. Set I bacteria showed a negative correlation with TNF-α and a positive correlation with IL-10, suggesting that FB091 uptake may have a protective effect against inflammation (Fig. 7). Conversely, Set II bacteria showed opposite results to Set I bacteria, supporting this finding. Therefore, FB091 administration may exert an anti-inflammatory effect.

In conclusion, FB091 supplementation may be closely related to the recovery and protection of liver health as well as the reduction of inflammation in the colon following alcohol intake.

## Discussion

Alcoholic liver disease (ALD) is a significant global health issue due to its severe impact on liver function and limited treatment options with substantial side effects. ALD is now the leading indication for liver transplantation and a primary cause of liver-related deaths. Traditional treatments, particularly corticosteroids, are effective in reducing inflammation but carry significant risks due to their immunosuppressive properties [13]. This study aimed to explore the potential of a new probiotic strain, *L. plantarum* FB091, as a safer alternative for managing ALD. Although many studies have demonstrated the benefits of probiotic administration in various diseases, relatively few have investigated their role in alleviating ALD through the gut-liver axis. In this study, an in vivo mouse feeding study and correlated gut microbiome analysis were performed to assess the recovery or protection effects of *L. plantarum* FB091 on ALD.

The in vivo mouse feeding trials indicated that alcohol and *L. plantarum* administration had no effect on water and feed intake, nor on body weight gain (Fig. 2). This finding is consistent with previous studies reporting no change in body weight in alcohol-fed mice [45]. However, histopathological analysis of liver and colon tissues revealed that alcohol administration increased hepatocellular ballooning and inflammatory cell infiltration in the liver, as well as structural damage in the colon (Fig. 3). Hepatocellular ballooning is a marker of severe liver cell injury in alcoholic liver disease, commonly associated with steatosis and inflammation [46]. This condition is exacerbated by the infiltration of inflammatory cells, leading to tissue damage and potentially progressing to fibrosis and cirrhosis [47]. Structural damage in the colon, such as disrupted crypt structure and mucosal barrier dysfunction, allows endotoxins to enter the bloodstream, increasing systemic inflammation [48]. However, FB091 administration reduced liver and colon structural damage, suggesting that FB091 helps prevent inflammation in these tissues (Fig. 3). While ATCC 8014 administration also mitigated liver damage, it did not prevent structural damage in the colon. This indicates that although both probiotic strains can reduce liver inflammation, only FB091 effectively protects the colon from structural damage.

To evaluate the effects of FB091 on liver damage and alcohol metabolism, three key biomarkers (AST, ADH, and ALDH) were measured. AST is a key indicator of liver injury, while ADH and ALDH are crucial enzymes in alcohol metabolism, converting ethanol to acetaldehyde and then to less toxic acetic acid. Elevated AST levels and impaired activities of ADH and ALDH are signs of alcohol-induced liver damage [49]. In the ALD+FB091 group, AST levels were reduced, and ADH activity increased compared to the ALD group, suggesting improved liver function and enhanced alcohol metabolism (Fig. 4A and 4B) with FB091 administration. However, the ALD+ATCC group showed no significant reduction in AST levels or increase in ADH activity. These findings suggest that FB091 provides a more effective protective effect against alcohol-induced liver damage compared to ATCC 8014, highlighting its potential therapeutic benefits.

Chronic alcohol consumption can disrupt gut barrier function, leading to increased translocation of endotoxins, which in turn triggers the production of inflammatory cytokines such as TNF-α, exacerbating both liver and colon inflammation [50]. To evaluate the immune regulation function of FB091, inflammatory cytokines in colon tissues were measured (Fig. 5). Pro-inflammatory cytokine TNF-α was elevated in the ALD group but reduced in the ALD+FB091 group. Additionally, anti-inflammatory cytokine IL-10 was decreased in the ALD group but increased in the ALD+FB091 group, indicating that *L. plantarum* FB091 exerts anti-inflammatory effects. This anti-inflammatory effect is supported by previous research showing that specific probiotic strains can interact with gut-associated lymphoid tissue (GALT) to influence regulatory T cells and dendritic cells, increasing the production of anti-inflammatory cytokines like IL-10 and suppressing pro-inflammatory cytokines like TNF-α, helping to restore gut barrier function and reduce systemic inflammation, thereby improving colon health damaged by alcohol [51].

To further understand the effect of *L. plantarum* FB091 administration in the ALD mouse model, gut microbiome analysis was conducted. The results showed significant alterations in the gut microbiota composition of the ALD+FB091 group compared to the ALD group. In the ALD group, the relative abundance of Cyanobacteria increased, indicating gut dysbiosis typically associated with alcohol-induced liver disease [52]. However, in the ALD+FB091 group, the administration of *L. plantarum* FB091 led to a reduction in Cyanobacteria compared to the ALD group. This shift in gut microbiota composition was comparable to the NC group, indicating that FB091 helps re-balance the gut microbiota composition affected by alcohol intake. Furthermore, the ALD+FB091 group exhibited higher abundance of beneficial bacteria, such as *Akkermansia* and *Lactobacillus*, which are known to enhance gut barrier function and exert anti-inflammatory effects [53]. Specifically, *Akkermansia* and *Lactobacillus* showed negative correlations with AST levels, suggesting a protective role against liver damage. These bacteria also correlated positively with IL-10 levels and negatively with TNF-α levels, suggesting their anti-inflammatory effects. Conversely, *Klebsiella* and *Streptococcus* showed opposite correlation patterns, supporting previous studies demonstrating their association with exacerbated ALD [54, 55]. *Klebsiella* promotes liver inflammation through the production of endotoxins like lipopolysaccharides (LPS), which penetrate the gut barrier and trigger immune responses in the liver, leading to increased production of pro-inflammatory cytokines such as TNF-α [54]. Additionally, *Streptococcus* can produce virulence factors like exotoxins and enzymes that degrade host tissues, contributing to liver inflammation [55]. Consequently, a high abundance of these bacteria in the gut microbiota is correlated with severe liver histopathology, liver inflammation, and fibrosis in ALD.

This in vivo ALD mouse model study demonstrated the recovery and protection effects of *L. plantarum* FB091 on liver and colon health from alcohol consumption. However, further human feeding trials and related clinical and omics analyses are necessary to evaluate the liver recovery and protection functions of *L. plantarum* FB091.
